# Pellets of proof: First glimpse of the dietary composition of adult odonates as revealed by metabarcoding of feces

**DOI:** 10.1002/ece3.3404

**Published:** 2017-09-14

**Authors:** Kari M. Kaunisto, Tomas Roslin, Ilari E. Sääksjärvi, Eero J. Vesterinen

**Affiliations:** ^1^ Zoological Museum Biodiversity Unit University of Turku Turku Finland; ^2^ Department of Ecology Swedish University of Agricultural Sciences Uppsala Sweden; ^3^ Department of Agricultural Sciences University of Helsinki Helsinki Finland

**Keywords:** 16S, cytochrome oxidase subunit I, damselfly, diet, dragonfly, fecal DNA, food web, Illumina MiSeq, Odonata

## Abstract

Recent advances in molecular techniques allow us to resolve the diet of unstudied taxa. Odonates are potentially important top‐down regulators of many insects. Yet, to date, our knowledge of odonate prey use is based mainly on limited observations of odonates catching or eating their prey. In this study, we examine the potential use of metabarcoding in establishing the diet of three adult odonate species (*Lestes sponsa*,* Enallagma cyathigerum,* and *Sympetrum danae*) at a site in southwestern Finland. To this purpose, we compared three different methods for extracting DNA from fecal samples: the Macherey‐Nagel Nucleospin XS kit, a traditional salt extraction, and the Zymo Research Fecal Microprep kit. From these extracts, we amplified group‐specific mitochondrial markers (COI and 16S rRNA) from altogether 72 odonate individuals, and compared them to comprehensive reference libraries. The three odonate species show major overlap in diet, with no significant differences between individuals of different size and/or gender, reflecting opportunistic foraging of adult odonates. Of a total of 41 different prey species detected, the most frequently consumed ones were Diptera, with additional records of six other orders. Based on our data, the best DNA extraction method is the traditional salt extraction, as it provides the most information on prey content while also being the most economical. To our knowledge, this is the first study to resolve the species‐level diet of adult odonates. Armed with the appropriate methodological caveats, we are ready to examine the ecological role of odonates in both terrestrial and aquatic food webs, and in transferring subsidies between these two realms.

## 1 INTRODUCTION

Recent advances in molecular techniques have opened up new ways for identifying prey species from fecal samples. In particular, they allow us to detect trophic links involving taxa the habits of which prevent us from efficient observations of direct feeding events (Clare, 2014; Roslin & Majaneva, 2016). Over the last few decades, ecologists have increasingly applied molecular tools to describing the diet of insectivore and even sanguinivore (blood‐feeding) mammals, spiders, birds, and many more taxa (Bobrowiec, Lemes, & Gribel, 2015; Deagle et al., 2005; Pompanon et al., 2012; Roslin & Majaneva, 2016; Symondson, 2002). In this context, a specific methodological challenge emerges for generalist insectivores where individual gut contents may contain many different prey items—as in such cases, the information content to be extracted from the assemblage of partly degraded DNA is much more complex than for a specialist predator (with solutions offered by e.g., Kruger, Clare, Symondson, Keiss, & Petersons, 2014; Paula et al., 2015; Pinol, San Andres, Clare, Mir, & Symondson, 2014; Vesterinen, Lilley, Laine, & Wahlberg, 2013; Vesterinen et al., 2016). Among air‐borne insectivores, bats have recently emerged as a particularly well‐studied group (Clare, 2014; Clare, Symondson, & Fenton, 2014; Clare, Symondson, & Broders et al., 2014; Emrich, Clare, Symondson, Koenig, & Fenton, 2014; Vesterinen et al., 2013, 2016), whereas the diet and ecological role of flying insect predators are next to unknown (but see Seifert & Scheu, 2012).

Insects in the order Odonata, including both dragonflies and damselflies, are a globally diverse group of insects with around 5,900 species described to date. Odonates are an important top predators in many aquatic and riparian ecosystems, thus representing both the aquatic and aerial environments and they are fairly well‐known due to decades of research (Corbet, 1999). Indeed, odonates have long been model organisms for ecological research, and form a highly promising taxon for future genomics focused research (e.g., Bybee et al., 2016; Córdoba‐Aguilar, 2009). Their role in global ecosystems is likely large, as odonates are common, large and remain predators throughout their life cycle (Askew, 2004). The extended larval stage is spent in water, and both diversity and biomass can be very high (e.g., Corbet, 1999; McCauley et al., 2008). Upon hatching, the adult odonate transfers to the terrestrial realm, thus moving biomass and energy from one habitat to another, and contributing to the predation pressure in a new environment.

However, despite that the role of the odonates in both aquatic and terrestrial ecosystems is likely to be large, the prey use of odonates is poorly documented, especially at adult stage of the life cycle. This is due to multiple constraints: for example, their prey species are usually small and thus hard to identify by observing them in mid‐air. Furthermore, visual prey observations of hunting odonates are likely to be biased toward large prey species. After successful hunting, odonates chew their prey thoroughly, which makes morphological identification of prey remnants from feces practically impossible. Fortunately, molecular tools—involving DNA extraction from predator remains, amplification of prey DNA by PCR, sequencing and identification through comparison to reference sequences (Clarke, Czechowski, Soubrier, Stevens, & Cooper, 2014; King, Read, Traugott, & Symondson, 2008; Pompanon et al., 2012; Roslin & Majaneva, 2016)—are not restricted by these aforementioned obstacles, and for the first time we are able to shed light on precise dietary composition of the odonates. Understanding the prey use of adult odonates is particularly important, as environmental conditions, for example, food shortage during the adult stage can reduce life span and fecundity, thereby reducing lifetime egg production and leading to numerical effects at the egg and larval stages (Stoks & Cordoba‐Aguilar, 2012).

The majority of recent theory about predator–prey interactions is based on the assumption that size is a key factor structuring these interactions (Brose, 2010; Schneider, Scheu, & Brose, 2012). In fact, a number of studies have shown that a significant portion of structural information within food webs can be predicted from body size alone (Stouffer, Rezende, & Amaral, 2011; Williams & Martinez, 2000). This is particularly prominent in aquatic systems where predation is largely limited by the size of the predator's gape—such that the larger a consumer is, the larger its gape and the larger its prey (Brose et al., 2006; Morgan, 1989). While large prey may require too much energy to capture, handle and consume, prey that are too small are not worth the energy invested to capture them (Svanback, Quevedo, Olsson, & Eklov, 2015). This should result in a unimodal relationship between predator and prey body size (Brose, 2010; Woodward, Ebenman, Emmerson et al., 2005; Woodward, Speirs, & Hildrew, 2005); in other words, predators of different size are expected to target prey within a different range, the mode of which should be higher with increasing predator size (Williams & Martinez, 2000). Diet generality may also increase with body size, allowing larger predators to exploit a wider range of prey (Gilljam et al., 2011).

To evaluate the potential for molecular techniques to describe the diet of odonates, we target three sympatric odonate species. Drawing on a comprehensive DNA barcode library of potential prey (the Finnish Barcode of Life, FinBOL; www.finbol.org), we use next‐generation sequencing techniques (DNA extraction followed by PCR and Illumina MiSeq sequencing) to answer the following questions: (1) How do methodological choices (DNA extraction techniques and choice of markers) affect our perception of prey use? (2) What prey taxa do these adult odonate predators feed on? and (3) do co‐occurring odonate species and sexes of varying size differ in their prey use? We predict, firstly, that methodological choices, especially the selection of PCR primers, will affect the results, with more variable gene regions resolving more prey taxa. Secondly, we expect odonates of different species and sex to differ in size, and this size variation to reflect into prey choice, with larger odonate predators consuming larger prey.

## 2 MATERIAL AND METHODS

### 2.1 Study species

To evaluate the potential for molecular, DNA‐based techniques based on locus‐specific amplification of gene regions to describe the diet of odonates, we target three odonate species, the northern bluet *Enallagma cyathigerum* (Charpentier, 1840) (Coenagrionidae), common spreadwing *Lestes sponsa* (Hansemann, 1823) (Lestidae), and black darter *Sympetrum danae* (Sulzer, 1776) (Libellulidae) into this study (images of the species in Fig. 4). The target species were chosen to represent locally common dragonfly and damselfly species that are phylogenetically divergent, and have different life history strategies while sharing the same habitat and overlapping phenology (Corbet, 1999; Dijkstra, 2006; Dijkstra & Kalkman, 2012). *Enallagma cyathigerum*, a Coenagrionidae species, overwinters as a larva and develops into the adult stage later than most damselfly species in Finland. Contrary to the majority of damselfly species in Finland, *E. cyathigerum* forages commonly on open areas, also above watersheds. The second focal species, *L. sponsa* belonging to Lestidae, overwinters at the egg stage in Finland, and develops rather fast through the larval stage during the summer. The adults are among the most common damselfly species flying in July—August. *Lestes* species hunt commonly near or inside dense vegetation. The third target species, *S. danae* belonging to Libellulidae, overwinters as an egg, develops quickly in the spring, and then hatches mainly in July. *Sympetrum danae* is by far the largest and strongest flyer of the focal species, foraging in open areas mainly by chasing its prey in mid‐air.

### 2.2 Study site and sample collection

Odonate samples were collected with aerial sweep nets. To remove variation between different foraging habitats and available prey, all our study samples were collected from one location in South West Finland (ETRS‐TM35FIN N: 671180; E: 24600): a freshwater wetland, surrounded by mosaic of arable land and cultural landscape. Altogether 25 odonate species have been observed around the study site after year 2014, indicating rather high species richness of this area (typical range of odonate diversity around typical watersheds is less than 20 odonate species in southern Finland; K. M. Kaunisto, personal communication). To maximize the comparability between samples and to reduce the effect of changes in the prey pool available, all samples were collected within 6 days (August 10–14, 2015) at a constant distance from the water body (5–8 m). The age of focal odonate individuals was determined by the stiffness of their wings and the coloration of their bodies (as described for genus *Calopteryx* in Plaistow & Siva‐Jothy, 1996). Only sexually mature individuals were included in this study. After being caught, sample individuals were placed into individual plastic Sarstedt 10‐ml tubes with a piece of moist paper towel added to avoid the dehydration of animals. Individuals were kept in the containers for 24 hr to allow complete defecation. All the fecal material (typically some 1–4 individual fragments of irregular size and shape) produced during this time was regarded as one sample. As a proxy for body size, we measured the length of hind wings and calculated average hind wing length for all the individuals. This metric has been previously shown to correlate well with body size (but see Schneider et al., 2012), and it is fairly easy to measure precisely. Thereafter, the feces were collected into Eppendorf tubes and frozen at −20°C until further analysis.

### 2.3 Molecular analysis

#### 2.3.1 Procedures for prevention of contamination

To minimize the risk contamination, we tried to adhere to the principles of ancient DNA processing as far as possible in the current laboratory. All the extraction steps were carried out in carefully cleaned laboratory space, using purified pipettes with filter tips. All the PCR's were carried out in a separate room, and no amplified DNA was transferred back to the pre‐PCR facilities. Negative controls containing all but template DNA were carried out for each PCR assay.

#### 2.3.2 DNA extraction using three different methods

Our dataset consisted of total 72 samples: 24 fecal samples for each three study species as equally distributed among females and males. The fecal material was not pretreated in any specific way prior to extraction, and the amount was so minimal (approximately 1 × 1 mm) that it was not practical to weigh the samples. The total set of samples was divided into three subgroups each consisting of 24 samples with equal representation of females and males of each study species (each subgroup contained four males and four females per species). One group was processed using ZR Fecal DNA MicroPrep (hereafter abbreviated as ZR; product nr D6012, Zymo Research, Irvine, California, U.S.A.), the second group using NucleoSpin^®^ Tissue XS Kit (abbreviation NS; product nr 740901, Macherey‐Nagel, Düren, Germany), and the third group with a traditional salt extraction method (abbreviation SE) (see Appendix S1 for detailed salt extraction protocol applied: Aljanabi & Martinez, 1997; Pilipenko, Salmela, & Vesterinen, 2012). We did not measure DNA concentrations, but expected them to be rather low due to the small amount of sample. Moreover, as we amplified both predator and prey DNA, the total DNA concentration as such would not be informative in any case. Thus, we used 1 μl of template DNA regardless of potential differences in the DNA concentrations.

#### 2.3.3 PCR and Illumina library construction

PCRs were prepared using the protocol of Clarke et al. (2014), with slight modifications related to the different indexing scheme as identified in Vesterinen et al. (2016). For this study, we used dual indexing designed for Illumina sequencing platform, and thus, both forward and reverse primers were tagged with different linkers, unique barcodes and Illumina‐compatible adapters (Shokralla et al., 2015). All the individual samples were tagged with a unique index combination. We chose to include the most common mitochondrial markers used for molecular identification of animals: *cytochrome oxidase subunit I* (hereafter abbreviated as COI) and 16S ribosomal RNA (16S) (Hebert, Cywinska, Ball, & DeWaard, 2003; Yang et al., 2014). The choice of COI region is natural, as most DNA barcoding to date has been carried out using this gene, resulting in millions of reference sequences available in the BOLD database (ref). The 16S region is the gene region second most commonly employed in DNA metabarcoding, and as this region is more conserved than COI, 16S primers usually amplify a larger set of taxa (Clarke et al., 2014). To amplify suitable fragments of approximately same lengths, we applied two primer sets—COI: primers ZBJ‐ArtF1c and ZBJ‐ArtR2c after Zeale, Butlin, Barker, Lees, and Jones (2011) and 16S: primers Ins16S‐1F and Ins16S‐1Rshort after (Clarke et al., 2014).

For this study, the PCR setup was further optimized as follows: for a reaction volume of 10 μl, we mixed 3.4 μl distilled water, 5 μl KAPA2G Fast MPX MasterMix (product nr KK5802, KAPA Biosystems, Wilmington, Massachusetts, USA), 0.3 μmol/L forward primer, 0.3 μmol/L reverse primer, and 1 μl DNA template. The PCR cycling conditions for COI were 3 min in 95°C, then 16 cycles of 30 s in 95°C, 30 s in 61°C (with the annealing temperature decreased by 0.5°C for each cycle) and 30 s in 72°C, then additional 24 cycles of 30 s in 95°C, 30 s in 53°C and 30 s in 72°C ending with 3 min in 72°C. For 16S, the cycling conditions were 3 min in 95°C, then 5 cycles of 15 s in 95°C, 30 s in 46°C and 15 s in 72°C, then additional 25 cycles of 15 s in 95°C, 30 s in 56°C and 15 s in 72°C. Then, 2.5 μl of PCR products of different primers were first pooled across samples and then cleaned using A'SAP clean kit (product nr 80350, ArcticZymes, Trømssa, Norway). All samples were used regardless of whether they produced a visible band on the gel used for checking. The second PCR used to attach adapters was implemented as in (Vesterinen et al., 2016), with minor modifications as follows: for a reaction volume of 12.5 μl, we mixed 6.25 μl KAPA HiFi HotStart MasterMix (product nr KK2602, KAPA Biosystems, Wilmington, Massachusetts, USA), 0.3 μmol/L forward primer, 0.3 μmol/L reverse primer, and 1.75 μl purified locus‐specific PCR product. The PCR cycling conditions were 4 min in 95°C, then 15 cycles of 20 s in 98°C, 15 s in 60°C and 30 s in 72°C, ending with 3 min in 72°C. Negative controls did not amplify in any assay. After tagging, 2 μl of each indexed sample was pooled together and purified using SPRI beads. Sequencing was performed on the Illumina MiSeq platform (Illumina Inc., San Diego, California, USA) by the Turku Centre for Biotechnology, Turku, Finland, using v2 chemistry with 300 cycles and 2*150 bp paired‐end read length. The pooled library was run together with other libraries using unique dual index combination for each sample.

#### 2.3.4 Sequencing output analysis and OTU identification

The sequencing run yielded 637,087 quality‐controlled paired‐end reads. The reads separated by each original sample were uploaded to CSC servers (IT Center for Science, www.csc.fi) for trimming and further analysis. Trimming and quality control of the sequences were carried out as follows. Paired‐end reads were merged and trimmed for quality using USEARCH version 9 (Edgar, 2010). Primers were removed using cutadapt version 1.11 (Martin, 2017). The reads were then collapsed into unique sequences (singletons removed), chimeras were removed, and reads were clustered into OTUs and mapped back to the original trimmed reads to establish the total number of reads in each sample using USEARCH version 9. Zero length OTUs do not practically differ from traditional clustering of OTUs, but the UNOISE algorithm performs better in removing chimeras, PhiX sequences and Illumina artifacts (Edgar & Flyvbjerg, 2015). Finally, our dataset consisted of 11,793 (COI) and 48,985 (16S) reads which were assigned to species. The OTUs were identified to species or—when species‐level determination could not be achieved—to higher taxa using BLAST (Altschul, Gish, Miller, Myers, & Lipman, 1990) and the Python script package “bold‐retriever,” version 1.0.0 (Vesterinen et al., 2016). Nearly all reads could be identified to at least order level and were thus retained for further analyzes. Rest of the reads were discarded: about 1% of COI reads were identified as Bacteria and plants; of 16S reads less than 1% matched human and other mammalian DNA. A detailed description of the bioinformatics applied is available from the authors upon a request.

Data on taxon‐specific size (body length of the prey taxa) were then extracted from literature or pictures from the BOLD database. In other words, prey taxa as per the taxonomic assignment of our sequences were used to estimate the body size range of the prey consumed, allowing the later testing of our explicit predictions regarding relationships between predator and prey size. From these tests, prey identified as “Hemiptera sp.” was omitted, as the size range within this compound taxon is too large to be informative.

### 2.4 Data analysis

To compare the size of odonates (hind wing length), we used ANOVA to model body size (predator hind wing length average) as a function of predator, sex and predator×sex. An equivalent model was fitted to data on prey size (prey body length) and to number of prey taxa detected (count of prey items in each sample).

In terms of prey used, we characterized the frequency of each prey taxon by its presence/absence at the level of individual odonate droppings. This approach was chosen as with PCR‐based approaches, the number of reads has been shown to carry little information about the original quantity of template DNA (Deagle & Tollit, 2007; Pompanon et al., 2012). Frequencies were calculated for each odonate species, for males and females and for different extraction methods.

To compare the effect of the gene region amplified, we calculated the number of prey items found in each sample separately for COI and 16S primers. We then used a Kruskal–Wallis Analysis of Variance procedure to compare the performance of each primer set in terms of the frequency distribution of prey items detected (Kruskal & Wallis, 1952). Likewise, we compared the total number of predator and prey reads among individual extraction methods. To further evaluate the performance of each DNA extraction method, we calculated the performance rate (as a percentage) by dividing the number of samples that produced at least some (prey or predator) reads with the number of samples used in the study. For this performance analysis of each extraction method, we used the read numbers remaining after quality control (see above). These performance metrics were calculated separately for each DNA extraction methods, odonate predator species, as well as for males and females within species.

We used ANOVA to model the number of samples that produced taxonomically assignable reads (as explained above) as a function of the different DNA extraction methods. For each extraction method, we also compared the ratio of predator versus prey reads. To visualize the trophic interactions structures resolved by the molecular data, we used package bipartite (Dormann, Fründ, Blüthgen, & Gruber, 2009) implemented in program R (R Core Team 2012). Semi‐quantitative webs were constructed for each odonate predator species, using proportional frequencies as explained above. To study the effects of body size, sex and predator species on variation in prey species composition, we conducted a permutational multivariate analysis of variance (PERMANOVA (Anderson, 2001) for presence/absence data, using 999 random permutations to assess statistical significance. PERMANOVA analysis was carried out using software R with package “vegan” (Oksanen et al., 2007). To visually compare odonate predator diets at the prey family level, we measured the proportion of the most frequent families (Diptera: Chironomidae, Sciaridae) consumed by each predator species.

## 3 RESULTS

### 3.1 How does the choice of DNA extraction method affect the results?

**Figure 1:**
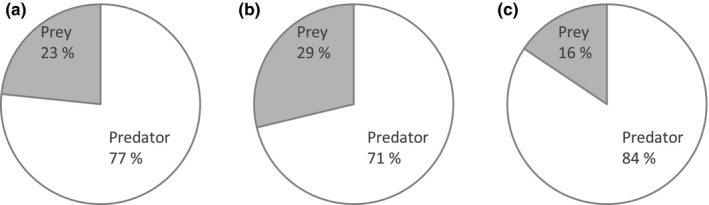
Efficiency of different extraction methods, reflected by the proportion of reads identified to prey taxa as retrieved by extraction with (a) the Macherey‐Nagel NucleoSpin XS kit, (b) the salt extraction method, or (c) the Zymo Research Fecal Microprep kit

**Figure 2:**
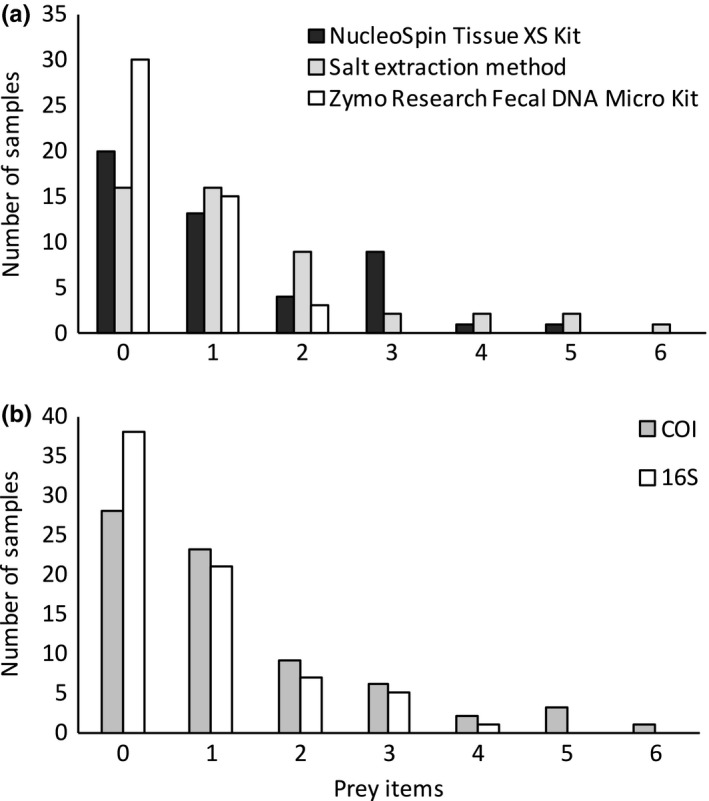
Prey identification success with different extraction methods or genetic markers. (a) Shown is the number of prey items identified per sample using each of the three DNA extraction methods, with (b) an equivalent graph for the two markers used: COI versus 16S. The zero prey items mean that the sample did not produce any sequences after bioinformatic pipeline. There was no significant difference in the frequency of prey detection between methods

Different extraction methods returned proportionally similar amounts of predator and prey reads (Fig. 1), although a very different amount of absolute target reads. The salt extraction method produced the highest number of reads (32,947), and also the highest number of prey taxa (27 distinct prey taxa). However, although commercial kits did not produce as many reads as did salt extraction, Nucleospin extraction resulted in almost as many prey taxa (26 prey species), as did the salt extraction method. Zymo Research performed the worse in both metrics, allowing the identification of only 11 different prey taxa. Success rates for each extraction method varied from 50% to 96% (Table 1). In terms of prey item detection rate per sample, we found no significant differences between different DNA extraction methods (Fig. 2a). Overall, COI primers produced a much lower number of predator reads than did 16S primers, but generally, the pattern of prey detection was similar between these two markers, as most of the samples only contained one prey item, while just a few contained more than four distinct prey species (Fig. 2b). The highest success rate was found when using salt extraction and 16S primers (95.8%), and the lowest success rate was observed with the Zymo Research kit and COI primers (50%; Table 1). On average, samples from females and males yielded similar success rates for both markers (COI: 61.1% vs. 66.7%; 16S: 94.4% vs. 97.2%; Table 1). For COI, most OTUs (33/48) and for 16S, less than half (15/37), were successfully identified to the species level or to an unequivocal higher taxonomic level (genus, family or order). In terms of reads (not OTUs), 87% of the trimmed COI reads and 98% of trimmed 16S reads offered a match in a database (BOLD and GenBank for COI; GenBank for 16S) and were retained for subsequent analysis. We found statistical differences between the DNA extraction methods and also between genetic markers in terms of how many prey items was retrieved per approach (Fig. 2a; Kruskal–Wallis 15.17, *p* = .0004) or markers (Fig. 2b; Kruskal–Wallis 4.43, *p* = .035). Labeled raw reads, read counts, and OTU data are available in the Dryad Digital Repository: https://doi.org/10.5061/dryad.5n92p.

**Table 1:** Success rate (%) in different strata of the data—that is, the number of samples producing sequence data (after processing by the bioinformatic pipeline) divided by the total number of samples in each group. COI and 16S refer to data retrieved by each primer pair. We found no significant difference between the number of successful samples for COI and 16S or different DNA extraction methods

	Nr of samples	COI %	16S %
Extraction method			
NucleoSpin Tissue XS Kit	24	66.7	95.8
Salt extraction method	24	75.0	100
Zymo Research Fecal DNA Micro Kit	24	50.0	91.7
Predator species			
* Enallagma cyathigerum*	24	79.2	100
* Lestes sponsa*	24	62.5	95.8
* Sympetrum danae*	24	50.0	91.7
Sex			
Females	36	61.1	94.4
Males	36	66.7	97.2

### 3.2 What prey species do odonates feed on and is there variation in the diet between odonate species and sexes?

**Figure 3:**
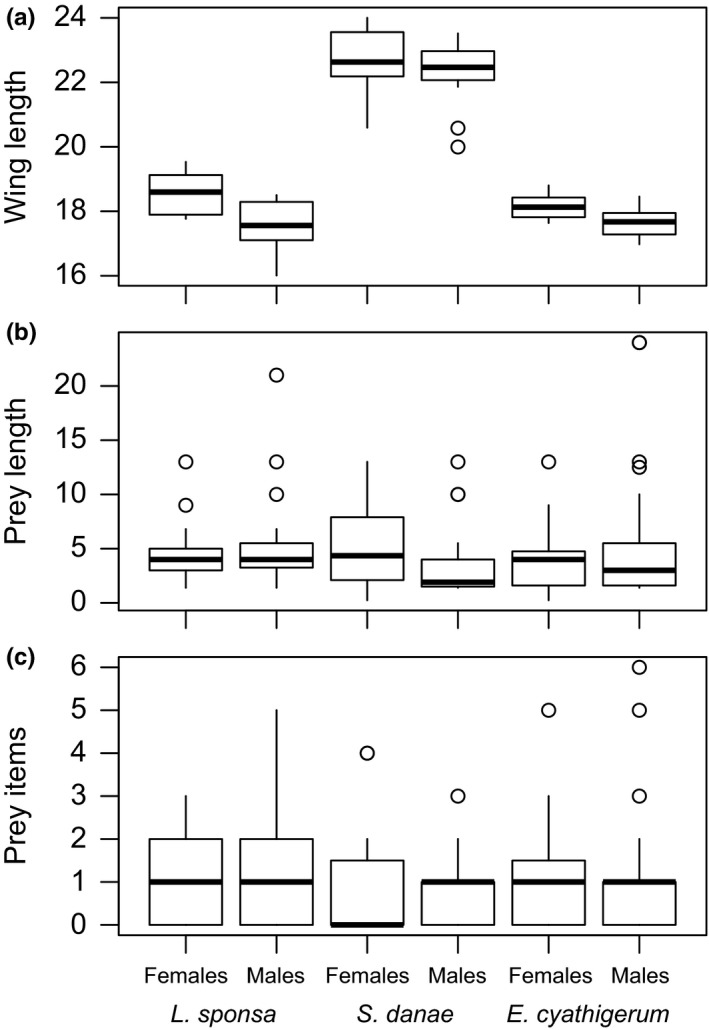
(a) Size of adult odonates (measured by the average of the length of the two hind wings), (b) size of prey taxa (measured by the body length of taxa), and (c) number of prey items per fecal sample, as resolved by predator species and sex

Altogether, we found DNA from 41 different prey taxa, representing 25 different families and seven orders (S2). Of these, 10 could only be assigned to family or higher taxa. Overall, the three odonate species differed in size, *S. danae* being significantly larger than the other two species (Fig. 3a; *F*_2, 69_=227.7, *p *= <.0001), with no difference among the two sexes (Fig. 3a; Sex *F*_1, 70_=1.36, *p* = .25; Sex × Predator *F*_2, 69_ = 1.02, *p* = .36). Nonetheless, the size of the prey consumed did not differ in size among either odonate species (Fig. 3b; *F*_2, 135_ = 0.05, *p* = .95), or sexes (Fig. 3b; Sex *F*_1, 139_ = 1.33, *p* = .25; Sex × Predator *F*_2, 135_ = 2.32, *p* = .10). Likewise, no significant differences in terms of the number of prey items detected per pellet were found between predator species (Fig. 3c; *F*_2, 139_ = 1.09, *p* = .34) or sexes (Fig. 3c; Sex *F*_1, 139_ = 0.07, *p* = .79; Sex × Predator *F*_2, 135_ = 0.19, *p* = .83).

**Figure 4:**
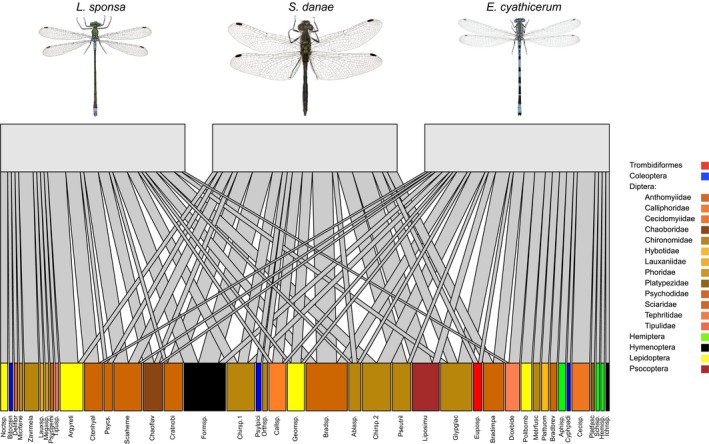
A semi‐quantitative food web of the odonate predator species and their prey, combining data from all extraction methods and both markers. The blocks in the upper row represent predators in each web and the blocks in the lower row the prey species. A line connecting a predator with a prey represents a detected predation event, and the thickness of the line represents the proportional frequency of each predation event. The web was drawn using method “cca” which minimizes the cross‐links between predators in R package “bipartite” (Dormann et al., 2009). Only the male pictures are shown, although the web is constructed from both sex diets. Pictures of odonates adopted from Norske Art databank under Creative Commons License (CC BY 4.0)

**Figure 5:**
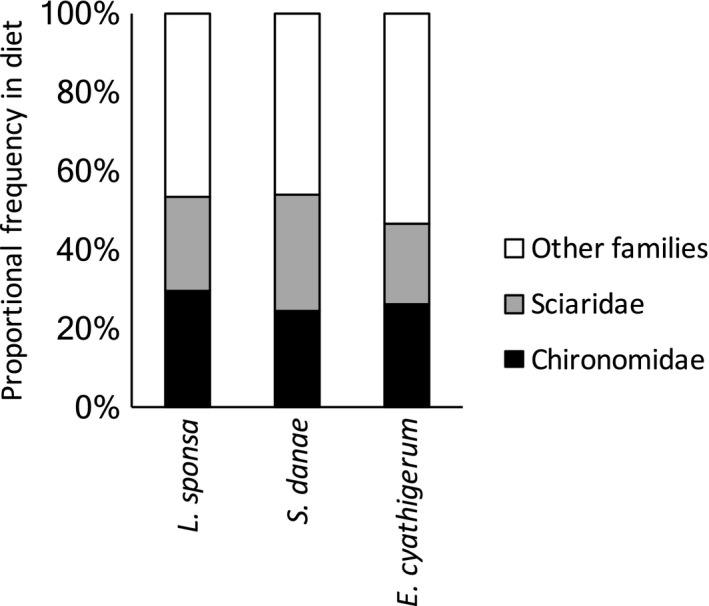
Prey use at the family level. Shown are the frequencies of the two most common families (Diptera: Chironomidae, Sciaridae) and of other families combined in the diet of each odonate species. EC = *Enallagma cyathigerum*, LS = *Lestes sponsa*, SD = *Sympetrum danae*

In terms of exact prey composition, different odonate species shared many prey species (Fig. 4). This dietary similarity was further confirmed by the multivariate analysis: after accounting for the effect of the body size, no significant differences remained between predators or sexes (ADONIS: *R* ^2^ = 0.04, *p* = .08; Table 2). The most common prey taxa were found in dipteran families Chironomidae (midges) and Sciaridae (dark‐winged fungus gnats), and when diet was compared at the family level, then the diet of the three predator species was indeed strikingly similar (Fig. 5).

**Table 2:** Permutational multivariate analysis of variance using Sørensen dissimilarity matrix of presence or absence of prey species in each sample. Terms were added sequentially to the model meaning that the significance of each term is evaluated against the background of terms above it. Predator body size was measured as the average hind wing length of each individual, with factor Predator referring to the species of dragonfly (three levels)

Predictor	SS	*F*	*R* ^2^	*p*
Predator body size	0.61	1.33	0.02	.146
Predator species	1.27	1.39	0.04	.076
Sex	0.53	1.19	0.02	.289
Predator x Sex	0.75	0.82	0.02	.781

## 4 DISCUSSION

To our knowledge, this is the first study to shed light on the complete species‐level diet of adult odonates. With the help of an extensive national barcode library, we were able to identify over forty prey taxa from the fecal samples. Of the 41 distinct prey identified, 28 were assigned to at least the genus level, and the rest (13) to a family (with one taxon only to the order) level.

### 4.1 Prey use by odonates

#### 4.1.1 Prey taxa consumed

Among the three odonate species studied here (*E. cyathigerum* (Coenagrionidae), *L. sponsa* (Lestidae), and *S. danae* (Libellulidae), the most commonly consumed prey order was Diptera. This group is undoubtedly one of the most abundant prey types available in the wet habitat where the study was conducted. It is also reported to be the most common prey taxon in previous odonate studies based on visual prey identification and sticky traps (e.g., Baird & May, 1997). Within Diptera, the most frequently observed taxa were families Chironomidae and Sciaridae. Indeed, these are some of the most abundant and diverse insect families in Finland, with approximately 700 and 340 species, respectively (Paasivirta, 2012, 2014; Vilkamaa, 2014). This also concur with previous study conducted with sticky traps in Japan, which found that at least 80% of individual prey items available for dragonfly *Mnais pruinosa* were small Diptera (Higashi, Nomakuchi, Maeda, & Yasuda, 1979).

We did not find statistical differences in the diet of the three focal odonates. Although the *S. danae* was significantly larger than other predator species, no significant difference was found in terms of prey size, detected prey items per sample, or prey assemblage. *S. danae* diet largely overlapped with that of the other predators, and included only two prey items unique to this species. In contrast, the other two predators (*L. sponsa* and *E. cyathigerum*) had nine and 11 unique prey species, respectively. At the level of prey families, the dietary patterns were highly similar between all three predators. Thus, all three species will likely exhibit an opportunistic hunting behavior, with slight differences in the exact prey species consumed reflecting chance events associated with the prey taxa encountered.

#### 4.1.2 Similarities to other air‐borne insectivores

Interestingly, the prey assortment detected in odonates was similar to that of bats living in similar habitats in the same region (Vesterinen et al., 2013, 2016). This similarity likely reflects joint features in habitat selection and foraging strategies, but also the high general availability of the main prey taxa (Diptera). Of the other diurnal species living in the same areas, the diet of birds is actually less explored by comparable techniques. Yet, results to date suggest that although birds undoubtedly consume large quantities of dipteran insects, they also catch more butterflies and moths (Lepidoptera), most probably at the larval stage (E. Vesterinen unpublished data). Thus, our methods offer a promising tool for assessing ecological similarities and dissimilarities among predator groups for which data have previously been hard to come by, and allow us to finally start mapping out the ecological significance of odonates. While the three focal odonate species differ in size and foraging tactics, there were no differences in the size of the prey they consumed. These results are rather surprising, as we a priori expected larger odonates to hunt larger prey species.

### 4.2 Methodological considerations

#### 4.2.1 Source of DNA

One of the issues complicating the interpretation of molecular information derived from food web studies is the source of DNA: whether it originates directly from the prey of focal predators or does it derive from lower steps in the food chain, a phenomenon commonly referred to as secondary predation (Boyer, Cruickshank, & Wratten, 2015; Sheppard et al., 2005). As the odonates are among the top predators of the insect world, they might consume many other predatory species, resulting in secondary predation. Furthermore, in case of parasite(oid)ism, it is possible that remnants of host species’ DNA could end up in these parasites and further to their predators. In this study, nonetheless, practically all of the prey items seemed to be species which are either herbivorous or do not feed as adults. Thus, the risk of false positives in the prey species list seems low in the current study. One exception for this in our results could be Trombidiformes, which include water mite species that commonly parasitize aquatic insects (e.g., Di Sabatino, Martin, Gerecke, & Cicolani, 2002). As our odonate species hunt close to water bodies, it is highly likely that they have consumed other insects that have been parasitized by the members of the order Trombidiformes and that this DNA is consequently represented in our results mainly via secondary predation. Another question is, whether the prey was caught by active hunting or scavenging for example from spider webs, behavior reported for helicopter damselflies by Ingley, Bybee, Tennessen, Whiting, and Branham (2012). However, even in such a case, it is not to be taken as a fault in the results or methods, but instead a challenge to be tackled by other methods, such as complementary direct observations.

Needless to say, contamination is also a real risk in any study dealing with tiny amounts of degraded DNA. Especially, when DNA is amplified, the unwanted DNA originating from contamination would amplify alongside resulting with false positives. In this study, we followed the procedures from our earlier works to cutting the risks to minimum (Vesterinen et al., 2013, 2016; Wirta et al., 2015). The greatest of caution needs to be taken not to introduce any sources of contaminating material to the laboratory while handling the samples. The amplification has to be done in a separate room (post‐PCR), and no amplified DNA should be taken back to the pre‐PCR facilities. The inclusion of negative control samples is a standard nowadays, but also positive “mock community” samples are used increasingly (Beng et al., 2016). The idea of mock communities is to add a sample containing a known mixture of potentially expected DNA and using the information from final data to interpret the molecular data quality. This is something to be built on in the future, although there is no easy way of standardizing the mock community approach between different studies. Despite the lack of positive controls with known DNA mixtures, we trust that we have succeeded in preventing the contamination and that what was found was actually eaten.

#### 4.2.2 Performance of different DNA extraction methods

Three different extraction methods were utilized in this study (Macherey‐Nagel Nucleospin XS Kit, salt extraction and Zymo Research Fecal Micro Kit). All these methods produced thousands of reads, which were subsequently assigned to various taxa. Salt extraction and Nucleospin retrieved more reads than Zymo Research kit, so in that sense they performed better. More importantly, salt extraction and Nucleospin enabled more prey taxa identifications than Zymo Research. Based on this, it can be concluded that of these three choices Nucleospin and salt extraction are recommended for molecular studies using odonate feces as starting material. The main technical differences between these methods are a) price (Salt extraction is substantially cheapest, around 0.1 euros per sample), b) the manpower required (Nucleospin is the least time consuming), and level of experience needed (commercial kits, such as Nucleospin, do not require that much earlier laboratory experience).

#### 4.2.3 Performance of different gene regions as markers

Both sets of primers chosen for our study targeted mitochondrial DNA, but amplified a different gene region. The COI region is the most common region applied in many different DNA barcoding studies as well as food web analyzes (Alberdi, Garin, Aizpurua, & Aihartza, 2012; Clare, Symondson, & Broders et al., 2014; Pastor‐Bevia, Ibanez, Garcia‐Mudarra, & Juste, 2014; Vesterinen et al., 2013, 2016; Wirta et al., 2015). COI typically offers high resolution in identifying the target taxa all the way to the species level (Hebert, Penton, Burns, Janzen, & Hallwachs, 2004; Hebert, Ratnasingham, & deWaard, 2003; Hebert, Cywinska et al., 2003), so it is a natural choice for any ecological research. However, some doubt has been cast over the use of the COI region in general, and in particularly over the use of so‐called mini‐barcode primers (especially ZBJ‐Art1c and ZBJ‐Art2c). The main criticism offered is that the primers may be biased, not amplifying arthropod taxa equally across the phylum (Clarke et al., 2014; Deagle, Jarman, Coissac, Pompanon, & Taberlet, 2014). The mitochondrial 16S rRNA region is more highly conserved than COI, offering a more suitable platform for generating widely generic primers. The complication is naturally that higher generality comes at the price of lower resolution of identification: 16S sequences usually cannot be attributed to species‐level taxonomy due both to less variable sites and to less populated reference libraries. In this study, we noticed a difference between identifications based on COI and 16S information: COI primers seemed to amplify only one of the odonate predators, *Enallagma cyathigerum*. This species was only identified from the stool of *E. cyathigerum*, suggesting either cannibalism or—more likely—a DNA origin in the cells lining the gut. On the other hand, 16S primers seemed to amplify all the odonate species examined, with every odonate predator species detected in the diet of all three predators. Taken at face value, this pattern may seem to suggest that the odonates are consuming each other. Furthermore, if these odonates were truly foraging on each other, the same pattern should have been visible in the COI reads, too, at least for the *E. cyathigerum* which was well amplified by the current primers. To add further resolution to future studies, we suggest that additional gene regions or multiple overlapping fragments from COI and 16S (allowing the reconstruction of longer sequences) may be amplified from the same samples. For the current cost‐effective library construction protocols, several complementary primers can be added without significantly increasing the total costs. PCR‐free methods offer another alternative (see Roslin & Majaneva, 2016), but offer additional problems and will be challenging for diet studies dealing with the current tiny amounts and degraded quality of prey DNA (Paula et al., 2015).

## 5 CONCLUSIONS

To our knowledge, this is the first study to shed light on the species‐level diet of adult odonates. Drawing on molecular, DNA‐based tools, we find that Odonata diet shows extensive overlap with previous records of bat diet and tentative records of bird diet, thus revealing major overlap in prey choice by dominant vertebrate groups. Different odonate species appear to overlap in diet, with no significant differences between individuals of different size and/or gender, reflecting opportunistic foraging of adult odonates. Based on the current study, we recommend using a traditional salt‐based method for the extraction of prey DNA from odonate fecal material. From an ecologic perspective, the current findings are partly conditional on a specific site and time. Thus, future studies are needed to evaluate the level of spatial and temporal variation in the dietary composition of odonates more generally. Our work identifies the tools needed for resolving such patterns. Equipped with the adequate methodological caveats, ecologists are now better prepared to establish the general role of odonates in terrestrial food webs as vehicles transporting subsidies between the aquatic and terrestrial realms.

## CONFLICT OF INTEREST

None declared.

## AUTHORS CONTRIBUTION

Kari M Kaunisto involved in original idea, field work, and writing the manuscript. Tomas Roslin involved in writing the manuscript and statistics. Ilari E Sääksjärvi: involved in writing the manuscript. Eero J Vesterinen: involved in laboratory analysis, statistics, and writing the manuscript.
