# Supplementary material for: Pellets of proof: First glimpse of the dietary composition of adult odonates as revealed by metabarcoding of feces
Source: Ecol Evol. 2017 Sep 14;7(20):8588–98. doi: 10.1002/ece3.3404 (PMC5648679; doi:10.1002/ece3.3404)
Supplement: Supplementary file 2 [file ECE3-7-8588-s002.doc]

**Pellets of proof: First glimpse of the dietary composition of adult odonates as revealed by metabarcoding of faeces**

Kari M. Kaunisto, Ilari E. Sääksjärvi, Tomas Roslin, and Eero J. Vesterinen

**Appendix S1: Extracting DNA from dragonfly faeces in 2.0 or 1.5 ml Eppendorf tubes**

A modified salt extraction protocol (after Aljanabi S, Martinez I 1997: Universal and rapid salt-extraction of high quality genomic DNA for PCR- based techniques. *Nucleic Acids Research*, **25**, 4692–4693).

1. Prepare lysis mix: **375 µl** of extraction buffer (see recipe on the next page) with **25 µl** Proteinase K (1250 ~µg/ml final concentration) per sample.

*- The lysis mix needs to dispensed immediately after mixing, so only prepare the amount that is used instantly (within 15 minutes).*

2. Dispense **400** **µl** of the lysis mix into a tube containing dragonfly faeces.

- *These tubes do not need to be labelled in great detail, as they will be discarded along the way. Numbering them on the lid is enough as long as there is detailed information about which number corresponds to which sample in your lab book.*

*- Small variation in the volumes has no effect, as long as the ratios are the same, for example: 200* *µl SE + 8 µl protK + 150 µl NaCl 🡪 300 µl supernatant + 300 µl isopropanol + 200 µl EtOH + preferred amount of sterile MQ-H_2_O*

3. Turn tubes upside-down a few times, shake them well and spin down.

4. Leave to digest at **+56 ºC for three hours**. Vortex occasionally during incubation.

- *The highly recommended alternative to incubating+vortexing is to digest the samples in a horizontal position in the shaker.*

5. Add **300 µl** of 6 M NaCl to the tube (this is 0.75x from the step 1 buffer volume). Vortex for 30 seconds.

6. Place the tube into a centrifuge and spin at 11 000 x *g* for 20 minutes. Then transfer **600 µl** of the clear supernatant to a **labelled 1.5 ml tube**.

- *During this long centrifuge step it is good to label the final tubes.*

- *These are the final tubes that the DNA will be stored in and should thus be labelled with* ***sample code(, species, date, and place).***

8. Add equal volume (~**600 µl**) of isopropanol to each tube. Mix by turning tube upside down a few times.

- Isopropanol may be pipetted into the empty fresh tubes just before

9. Place tube into -20 ºC for one hour.

*- One hour is minimum time, can be kept in freezer for longer, e.g. overnight*

10. Centrifuge tubes at (+4 ºC and) 14 000 x *g* for 20 minutes.

11. Pour out isopropanol and wipe tubes. Be careful not to discard the DNA pellet.

12. Add **~1000 µl** of ice cold 70 % ethanol to wash the pellet and to remove isopropanol residues.

13. Centrifuge tubes at +4 ºC and 14 000 x *g* for 10 minutes.

14. Pour out ethanol (be careful not to discard the DNA pellet) and leave tubes to dry at +56 ºC until dry.

15. Add **20 µl** of sterile MQ-H_2_O to each tube and leave at room temperature for 1 hour.

16. Store at -20 ºC.

**RECIPES**

**Note!** The calculations are based on stock solution concentrations commonly available.

If you have different stock concentrations, calculate yourself: **C_1_ * V_1_ = C_2_ * V_2_**

**Extraction Buffer:**

*Stock concentrations*

5 M NaCl

1 M Tris-HCl pH 8.0

0.5 M EDTA pH 8.0

20 % SDS

*Final concentrations*

0.4 M NaCl

10 mM Tris-HCl pH 8.0

2 mM EDTA pH 8.0

2 % SDS

*Preparation of working buffers*

*For 250 ml final volume, take a sterile 250 ml glass bottle, and add*

20 ml NaCl

2.5 ml Tris-HCl pH 8.0

1 ml EDTA pH 8.0

2.5 ml SDS

224 ml MQ-H_2_O (up to 250 ml)

*For 1 litre final volume, take a sterile 1000 ml glass bottle, and add*

80 ml NaCl

10 ml Tris-HCl pH 8.0

4 ml EDTA pH 8.0

10 ml SDS

896 ml MQ-H_2_O (up to 1 litre)

**6 M NaCL (saturated) solution:**

Molar weights: Na ≈ 23 g; Cl ≈ 35.44; NaCl ≈ 58.44

1M NaCl in 1 litre = 58.44 g NaCl filled up to 1 liter MQ-H_2_O

6M NaCl in 250 ml = 58.44 g * (6M/4) ≈ 87.66 g NaCl filled up to 250 ml MQ-H_2_O

**Pellets of proof: First glimpse of the dietary composition of adult odonates as revealed by metabarcoding of faeces**

Kari M. Kaunisto, Tomas Roslin, Ilari E. Sääksjärvi, and Eero J. Vesterinen

**Appendix S2: Prey taxa observed in the study**

**Table S2.1.** Frequency (proportions out of n=72 droppings) of the prey species identified for each odonate species using markers targeting the COI or the 16S region.

| **Higher taxon** | **Prey species** | *Enallagma cyathigerum* | | *Sympetrum danae* | | *Lestes sponsa* | |
| --- | --- | --- | --- | --- | --- | --- | --- |
|  |  | COI | 16S | COI | 16S | COI | 16S |
| **Class Arachnida** |  |  |  |  |  |  |  |
| **Trombidiformes** |  |  |  |  |  |  |  |
| Eupodidae | sp. | 0.04 |  | 0.08 |  |  |  |
| **Class Insecta** |  |  |  |  |  |  |  |
| **Coleoptera** |  |  |  |  |  |  |  |
| Chrysomelidae | *Psylliodes picinus* |  |  | 0.08 |  |  |  |
| Scirtidae | *Cyphon padi* | 0.04 |  |  |  |  |  |
| Zopheridae | *Bitoma crenata* |  |  |  |  |  | 0.04 |
| **Diptera** |  |  |  |  |  |  |  |
| Anthomyiidae | *Delia florilega* |  |  |  |  | 0.06 |  |
| Calliphoridae | sp. |  | 0.04 |  | 0.09 |  | 0.04 |
| Cecidomyiidae | sp. | 0.22 |  |  |  |  |  |
| Chaoboridae | *Chaoborus flavicans* | 0.04 |  | 0.08 |  | 0.18 |  |
| Chironomidae | *Ablabesmyia sp.* | 0.04 |  | 0.08 |  | 0.06 |  |
|  | sp. |  | 0.08 |  | 0.09 |  | 0.13 |
|  | *Chironomus sp.* |  | 0.17 |  |  |  | 0.17 |
|  | *Glyptotendipes glaucus* | 0.17 |  | 0.17 |  | 0.12 |  |
|  | *Metriocnemus fuscipes* | 0.09 |  |  |  |  |  |
|  | *Microchironomus tener* |  |  |  |  | 0.06 |  |
|  | *Orthocladiinae sp.* |  |  | 0.08 |  |  |  |
|  | *Pseudosmittia trilobata* | 0.04 |  | 0.25 |  |  |  |
|  | *Zavrelimyia melanura* |  |  |  |  | 0.24 |  |
| Hybotidae | *Platypalpus tuomikoskii* | 0.09 |  |  |  |  |  |
| Lauxaniidae | sp. |  |  |  |  |  | 0.04 |
| Phoridae | *Megaselia sp.* |  |  |  |  | 0.06 |  |
| Platypezidae | *Platypeza fasciata* |  | 0.04 |  |  |  |  |
| Psychodidae | *Psychoda gemina* |  |  |  |  | 0.06 |  |
|  | sp. |  |  | 0.08 |  | 0.06 |  |
| Sciaridae | *Bradysia brevispina* | 0.09 |  |  |  |  |  |
|  | *Bradysia impatiens* | 0.13 |  | 0.17 |  |  |  |
|  | *Bradysia sp.* |  | 0.13 |  | 0.18 |  | 0.13 |
|  | *Cratyna nobilis* |  |  | 0.25 |  | 0.06 |  |
|  | *Ctenosciara hyalipennis* | 0.04 | 0.04 |  |  | 0.06 | 0.13 |
|  | *Sciara hemerobioides* | 0.04 |  | 0.17 |  | 0.24 |  |
| Tephritidae | *Dioxyna bidentis* | 0.13 | 0.04 |  |  | 0.06 |  |
| Tipulidae | Pedicia sp. |  |  |  |  |  | 0.04 |
| **Hemiptera** |  |  |  |  |  |  |  |
| Aphididae | *Aphis sp.* | 0.09 |  |  |  |  |  |
|  | *Schizolachnus pineti* | 0.04 |  |  |  |  |  |
|  | sp. | 0.04 |  |  |  |  |  |
| **Hymenoptera** |  |  |  |  |  |  |  |
| Formicidae | *Harpagoxenus sublaevis* |  | 0.08 |  | 0.18 |  | 0.17 |
| Ichneumonidae | sp. | 0.04 |  |  |  |  |  |
| **Lepidoptera** |  |  |  |  |  |  |  |
| Argyresthiidae | *Argyresthia retinella* |  |  | 0.08 |  | 0.29 |  |
| Geometridae | *Idaea straminata* | 0.04 |  | 0.17 |  | 0.06 |  |
| Noctuidae | Arctiinae sp. |  |  |  |  | 0.12 |  |
|  | *Polia bombycina* | 0.13 |  |  |  |  |  |
| **Psocoptera** |  |  |  |  |  |  |  |
| Liposcelididae | *Liposcelis simulans* |  | 0.08 |  | 0.18 |  |  |
